# Impact of menstrual cycle or combined oral contraception on elite female cyclists' training responses through a clustering analysis of training sessions

**DOI:** 10.3389/fspor.2024.1307436

**Published:** 2024-02-29

**Authors:** Hugo Carlin, Marine Dupuit, Florent Storme, Tom Chassard, Alice Meignié, Iris Sachet, Emanuel Brunet, Jean-François Toussaint, Juliana Antero

**Affiliations:** ^1^ Institut de Recherche bioMédicale et d'Epidémiologie du Sport (IRMES, UPR7329), INSEP (Institut National du Sport, de l'Expertise et de la Performance), Paris, France; ^2^ Fédération Française de Cyclisme (FFC), Saint Quentin en Yvelines, France; ^3^ Centre d'Investigations en Médecine du Sport—CIMS, Hôpital Hôtel-Dieu, AP-HP, Paris, France; ^4^ Université Paris Cité, Paris, France

**Keywords:** menstrual cycle, oral contraceptive pills, athletes, women, training classification, clustering, training responses, cycling

## Abstract

**Objectives:**

(i) To classify training sessions of elite female cyclists according to an intensity index based on a longitudinal follow-up using multiparametric data collected *in situ* (ii) to measure the effect of estimated menstrual cycle (MC) phases and oral contraceptive pills (OC) phases on the athletes' training responses on each type of training identified.

**Method:**

Thirteen elite French cyclists were followed up over 30 months and 5,190 training sessions were collected and 81 MC/OCs full cycles analyzed. Power sensors and position devices captured training data *in situ*, which was summarized into 14 external load variables. Principal Component Analysis and K-means clustering were used to identify cycling sessions according to an intensity load index. The clusters were then verified and categorized through the analysis of heart rate and rate of perceived effort. A calendar method was used to estimate 3 phases of the MC: menstruation, mid-cycle phase (MP) and late-cycle phase (LP). Two phases were defined among monophasic OC users: pills' taking/withdrawal.

**Results:**

Four main types of training effort were identified: Intensive, Long, Medium and Light. In the MC group (*n* = 7; 52 cycles), the intensity index is 8% higher during the mid-cycle (vs. menstrual phase, *p* = 0.032) in the Intensive effort sessions. No differences were observed in Long, Medium or Light effort, nor between the phases of pills' taking/withdrawal among OC users.

**Conclusion:**

The clustering analyses developed allows a training classification and a robust method to investigate the influence of the MC/OC *in situ*. A better training response during the mid-cycle when the sessions are the most intense suggest an impact of the MC when the athletes approach their maximal capacity.

## 1 Introduction

Menstrual cycle (MC) of female athletes has been suggested to impact their training responses (1) with a possible performance impairment during the menstrual phase (2). However, studies investigating the MC impact on female athletes' training responses are limited and lack robust methodological analyses such as longitudinal studies (3, 4). It remains unclear which training variables are influenced by the different phases of the menstrual cycle. There is also a lack of studies providing conclusive findings on athletes with hormonal contraception (5), including combined oral contraceptive pills (OC) which are commonly used in athletic population (6).

The impact of MC or OC phases has been suggested to be minor (7). Hence, it is crucial to rely on longitudinal follow-up over several months with multiparametric daily data collection, preferably *in situ*, to expect to distinguish the impact of MC or OC on athletes' training responses (5). Elite cyclists provide a robust population for such analysis, considering the massive data available from the regular use of power sensors during training sessions. Yet, the challenge lies in analyzing training datasets where each session has specific goals (e.g., recovery, speed, endurance) involving therefore different intensity effort levels. Thus, it is essential to differentiate the types of training sessions to investigate the impact of MC or OC on sessions with similar intensity demands. Hence, the development of a training type classification model prior to cycles analyses is necessary.

While training volume has been traditionally used to quantify training load (8, 9), this variable alone is not sufficient to accurately distinguish between different training sessions demands (10). Intensity, referring to the magnitude of measurable variables such as power, force or speed (11), plays a crucial role in training data analysis, since it is a good proxy of training performance (10, 12). However, quantifying training intensity is challenging because it depends on multiple other parameters (e.g., altitude, average power, maximal speed). No previous studies have attempted to identify cycling training types based on an intensity demand index.

Therefore, we aimed (i) to classify the training sessions of elite female cyclists based on an intensity index using multiparametric data collected *in situ*, to (ii) measure the effect of the MC and OC phases on the athletes' training responses on each training type identified, based on a longitudinal follow-up.

## 2 Methods

A total of 13 elite French cyclists, tier 4 and 5 according to the international classification (13) with a natural menstrual cycle or using monophasic pills, volunteered to participate in this study. Prior to their participation, all athletes received detailed information regarding the purpose of the study and provided their informed consent. A prospective follow up of their MC or OC length and regularity took place during 30 months, from April 2020 to September 2022. The posteriori inclusion criteria was an absence of hormonal contraception change and a regular cycle in the MC group, defined as a variation of fewer than 7 days in the length of their last 3 cycles and a cycle length between 22 and 35 days (14). Training data, such as power, speed, heart rate, altitude, were collected *in situ* during the 30 months of follow up and more 34 months retrospectively since June 2017 for a robust clustering classification.

### 2.1 Training data collection and management

Power sensors and global positioning systems (GPS) devices were employed to collect the training data. Power sensors utilized extensometry, which involves measuring deformations, to determine power output. Power is calculated by multiplying the torque, which is measured through the constant deformation of calibrated bike components, by the angular velocity. The sensor on the pedal and gear mechanism determines the measurement of angular velocity and power, with pedaling rate recorded from the pedals. GPS devices were used to analyze the athletes' movement and track their speed, position, altitude and movement.

Common measures in external and internal load monitoring were recorded every training second for external load variables—power, cadence, speed, altitude, torque, distance, time—and internal load variables—heart rate, and rating of perceived effort (RPE) (15).

An aggregated summary of each session was created by combining 14 external load variables at total: (i) the total training time, (ii) the total distance, (iii) the average speed, (iv) the average power, (v) the average cadence, (vi) the average torque, (vii) the positive elevation gain (sum of the distance in increasing altitude), (viii) the negative elevation loss (sum of the distance in decreasing altitude), (ix) time in positive elevation gain (sum of time in increasing elevation), (x) time in negative elevation loss (sum of time in decreasing elevation), (xi) the average power in positive elevation gain (average of power in increasing elevation), (xii) average speed in positive elevation gain (average of speed in increasing elevation), (xiii) the Intensity Factor (IF), and (xiv) the training stress score (TSS). IF quantifies the intensity of a cycling session based on power and provides an indication of the difficulty relative to the athlete's fitness level. It is calculated by dividing the normalized power by the functional threshold power (16). TSS, also known as “Coggan's Load” (16), is a specialized measure of external load for cycling that evaluates the intensity of physiological stress during a biking session and the overall difficulty of the session. TSS is calculated using normalized power, IF and duration of activity.

Regarding the internal load variables, the heart rate was collected through heart rate monitors. The RPE, was collected after each training session using and application previously described (17), according to the BORG scale CR10 (18).

Cyclists used sensors from different brands, but all data were standardized into a homogeneous format, with the same load variables and units. To enable easy comparison among cyclists, considering individual variations, and to ensure comparability of results, a normalization process was conducted on all studied variables for each athlete. This normalization procedure rescaled the values between 0 (representing the smallest value) and 1 (representing the highest value). As the min-max scaling could be sensible to outliers, abnormal values (± 2 × standard deviation) were excluded.

The training planning and periodization were conducted by the coaches throughout the period, independent of this study, aligning with the cyclists' requirements to achieve readiness according to their competition goals. The coaches were blinded to the cyclists’ phases.

### 2.2 Menstrual status monitoring and phases determination

The menstrual status monitoring relied on a menstrual diary, which was completed daily using the same application for RPE collection to determine the beginning and the end of the menstruation phase or the pills withdrawal phase (17, 19). To investigate the influence of estimated MC and OC phases on training, different phases were defined depending on athletes' menstrual status (19).

For cyclists in the MC group, we estimated the ovulation day for each cycle period using a linear regression model based on each cyclist cycle length through the follow up duration, showing a high accordance with the real ovulation date in previsous studies (20). Then the cycle was divided into 3 calendar phases: the menstrual phase, comprising the bleeding days; the mid-cycle phase (MP), from the last day of menstrual bleeding to the estimated ovulation day; and late-cycle phase (LP), starting after the MP until the day prior to the beginning of the next menstrual bleeding.

For OC athletes, we divided their cycle into two phases: a withdrawal phase (WP) of 7 days (i.e., placebo, pause) and an active pill phase (APP) of 21 days (19).

### 2.3 Data analysis

To identify the most discriminating variables of the cyclists' effort, we conducted an initial principal component analysis (PCA) to represent all the variables from each training session in a coordinate system defined by two axes (21) based on all the training data collected since June 2017.

The clustering method is then employed to group similar elements together, thereby revealing hidden patterns and classifying training sessions. This is an unsupervised learning approach, characterized by the analysis and grouping of unlabeled data. We selected the K-means algorithm (22), which facilitates the grouping of cycling sessions into multiple classes based on the Euclidian distance between vectors representing the different sessions. The vectors are composed of the first two dimensions of the PCA. K-means algorithm is an iterative process comprising two steps: (1) each training session is assigned to the closest of the k centroids based on the Euclidean distance, thereby forming a cluster, and (2) new centroids are computed as the barycenter of each cluster. Initially, the centroids are randomly generated, and the process continues until the centroids become stable, either reaching a predetermined threshold or a predefined number of iterations. The Elbow method was utilized to determine the optimal number of clusters (23).

Once the training sessions have been classified into k clusters, we verified that each cluster represents a distinct type of training based on the first two principal components obtained from PCA. These distinctive characteristics were then used to label the clusters. Then, we used average power, average speed, heart rate, RPE, training time and distance, to verify if these parameters significantly varied among the identified clusters and validate the main characteristics of such clusters. Finally, for interpretation and validation of consistency within the clusters of data, we used the Silhouette method (24).

To test for significant differences across the MC or OC groups within each cluster, we employed the Wilcoxon test (25). A confidence interval of 95% was determined using the LOcally Estimated Scatterplot Smoothing (LOESS) method.

To establish a significant difference across the MC or OC phases in each cluster we selected a threshold smaller than 0.05 corresponding to a 95% confidence level.

### 2.4 Ethics

The conducted investigations adhered to the code of ethics outlined by the World Medical Association (Declaration of Helsinki) and received approval from the Institutional Ethics Committee (IRB00012476-2022-03-11-206). The data collection process complied with the General Data Protection Regulation (2016/679) implemented in the European Union and received a certificate of compliance from the Commission Nationale Informatique et Libertés (CNIL - 2221532 v0).

## 3 Results

### 3.1 Study population

A total of 5,190 training sessions of 13 professional female cyclists (26.2 ± 3.7 years), were collected over the period between June 2017 and September 2022 and included in the training classification. Among these cyclists, 12 (26.6 ± 3.7 years, 57.2 ± 7.6 kg, 165.5 ± 6.9 cm) met the posteriori inclusion criteria for regular cycles: 7 had natural MC and 5 used OC monophasic pills. Overall, 81 full cycles were analysed, corresponding only to the menstrual cycles that remained regular throughout the entire follow-up period. On average, each athlete had 399 ± 124 training sessions with all the 14 external load variables recorded per session available for analysis.

**Figure 1:**
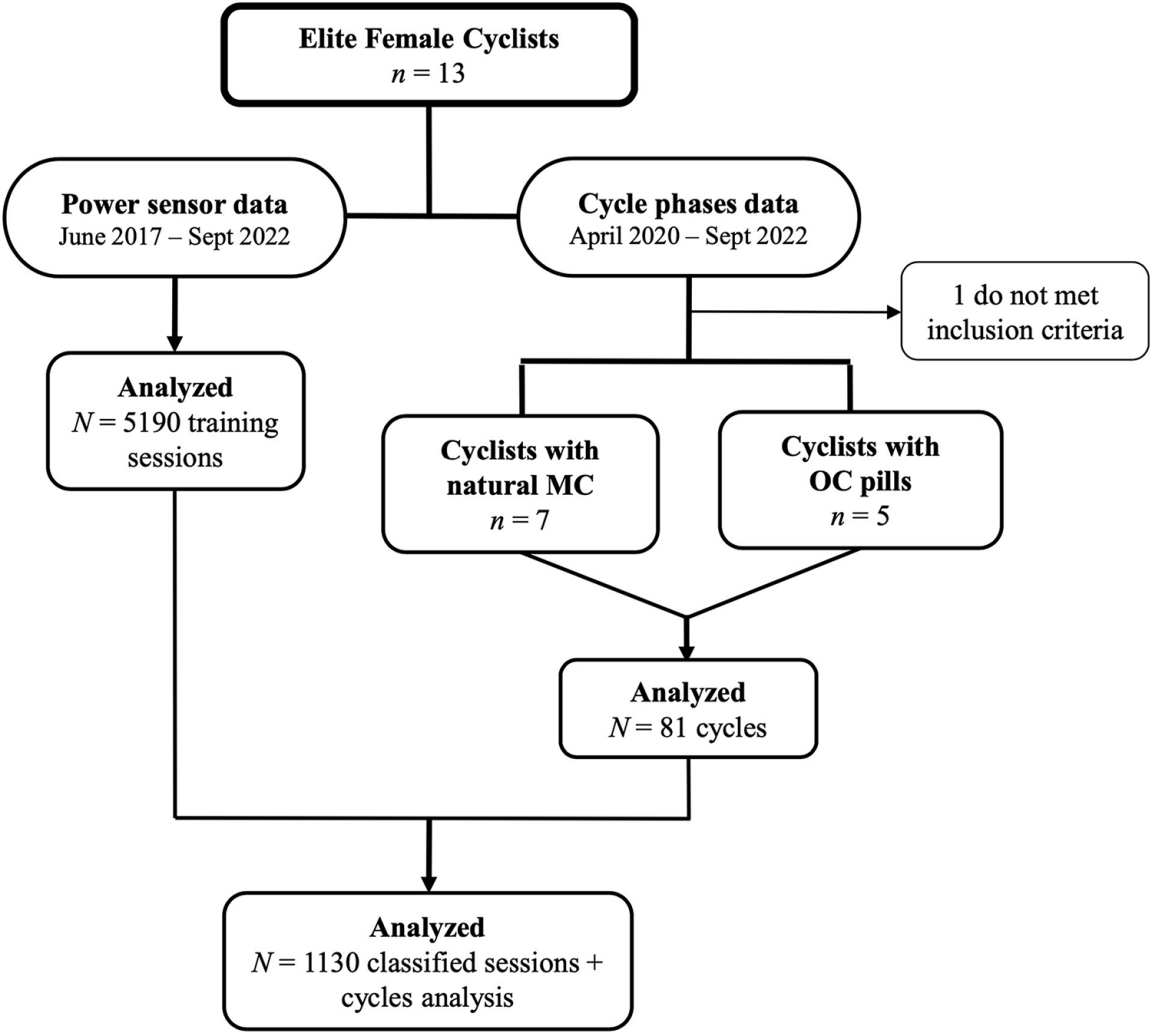
Data management flowchart. MC, menstrual cycle; OC, oral contraceptive.

By combining the training type data with the cycle-related data from April 2020 to September 2022, we obtained 1,130 complete observations (Figure 1).

### 3.2 Principal component analysis (PCA)

**Figure 2:**
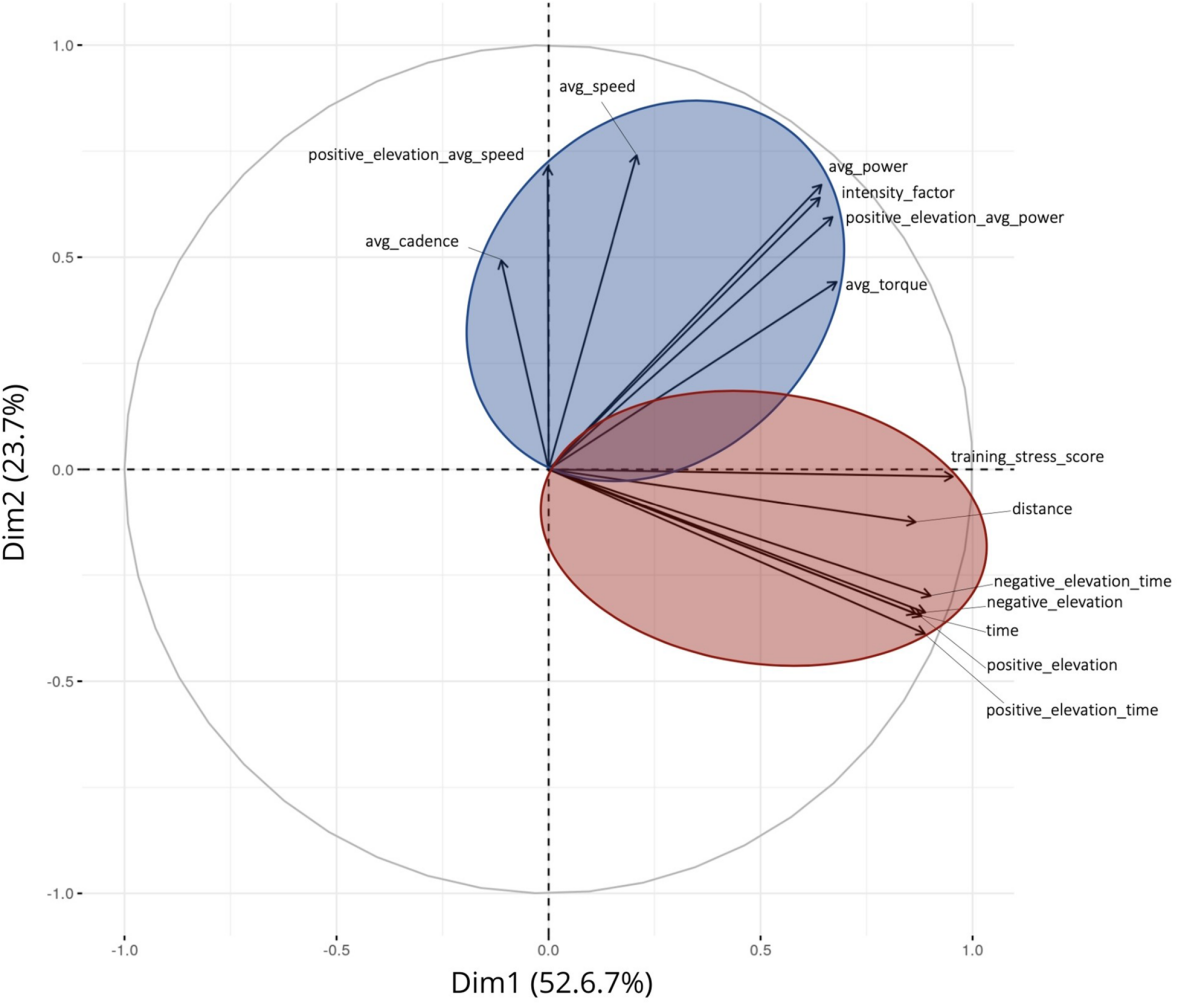
Principal component analysis of the 14 external load variables collected through power sensors and position devices among 5,190 training sessions of professional female cyclists. Two-axis were identified, the vertical one defining the mostly intensity-driven variables, regrouped in the blue area and the horizontal one, defining the mostly volume-driven variables, regrouped in the red area. Avg, average.

The PCA revealed two distinct groups of variables that form two axes in a system based on the cycling sessions (Figure 2). These two synthetic variables capture 76.3% of the overall dataset variability. The first variable, constructed through a linear composition of average cadence, average speed in positive elevation gain, average speed, average power, IF, average power in positive elevation gain and average torque, contributes for 52.6% of the total variance. The second variable, which incorporate TSS, total distance, time in negative elevation, total time, negative elevation loss, positive elevation gain and time in positive elevation, contributes to 23.7% of the variance. Based on their composition, the first principal component axis of the PCA is associated with training Volume, while the second component relates to training Intensity. The different training sessions are projected onto this Volume-Intensity pairing. The position of each session in this reference system serves as the basis for cluster labels.

### 3.3 Clustering

**Figure 3:**
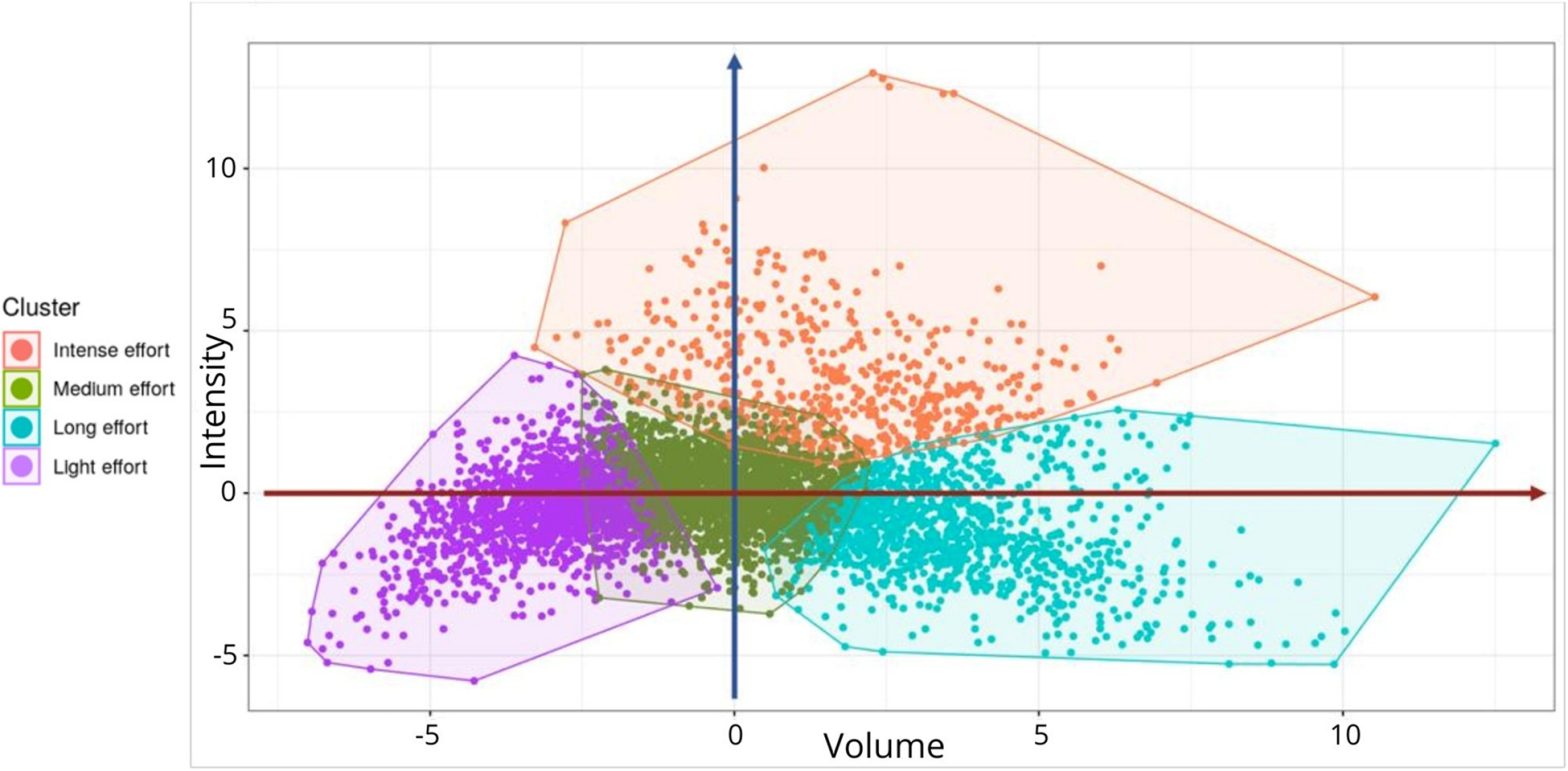
Clustering results of the four types of training identified illustrated by each colored area. Each point represents one training session (*n* = 5,190). The vertical blue arrow represents the intensity-axis. The horizontal red arrow represents the volume-axis. Further from the center, higher the Volume-Intensity index.

Four distinct clusters were identified (Figure 3), effectively separating training types with the most similarities. These clusters represent similar demands when considering the combined variables of the Volume-Intensity pairing. Clusters were categorized as: intensive effort (high intensity index/medium volume index), long effort (low intensity index/high volume index), medium effort (medium intensity index/medium volume index), light effort (low intensity index/low volume index). Among the training sessions monitored, 518 (=10%) were classified as intensive effort, 2,095 (=40%) as medium efforts, 1,126 (=22%) as long efforts, and 1,451 (=28%) as light efforts. The mean Silhouette score was 0.35 and 4.1% of training sessions had a negative score.

**Figure 4:**
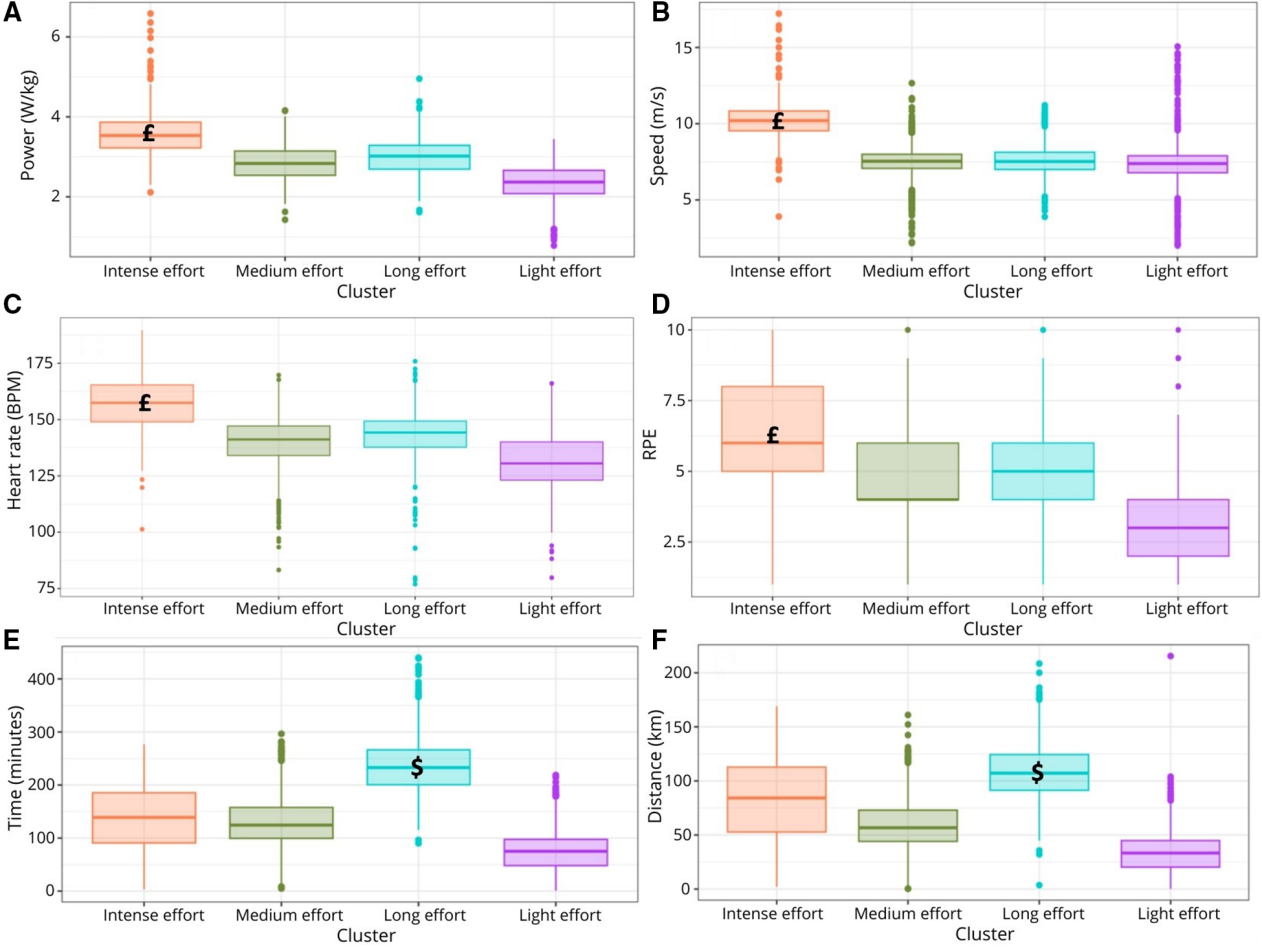
Boxplot of the average power (**A**), average speed (**B**), heart rate in beats per minute (BPM) (**C**), rate of perceived exertion (RPE) (**D**), training time (**E**) and training distance (**F**) by clusters. £: *p* < 0.05 Intense effort vs. other clusters; $: *p* < 0.05 Long effort vs. other clusters.

The intensive effort cluster displayed significantly higher average power (*p* < 0.0001, Figure 4A), average speed (*p* < 0.0001, Figure 4B), heart rate (*p* = 0.0064, Figure 4C) and RPE (*p* = 0.0004, Figure 4D) among the clusters. The long effort cluster exhibited significantly higher training time (*p* = 0.0013 Figure 4E**)** and training distance (*p* = 0.0016, Figure 4F) compared to the others three clusters.

### 3.4 Cycle phases analysis

Among MC athletes (cycle's length 29.1 ± 3.0 days), 144 (20.2%) training sessions occurred during the menstruation phase (5.3 ± 1.7 days), 285 (40%) during the mid-cycle phase (11.8 ± 2.2 days), and 284 (39.8%) during the late-cycle phase (12.1 ± 1.0 days). For OC users, 85 (20.4%) training sessions occurred during the 7 days of pills’ withdrawal phase, and 332 (79.6%) during the 21 active pills’ phase.

**Figure 5:**
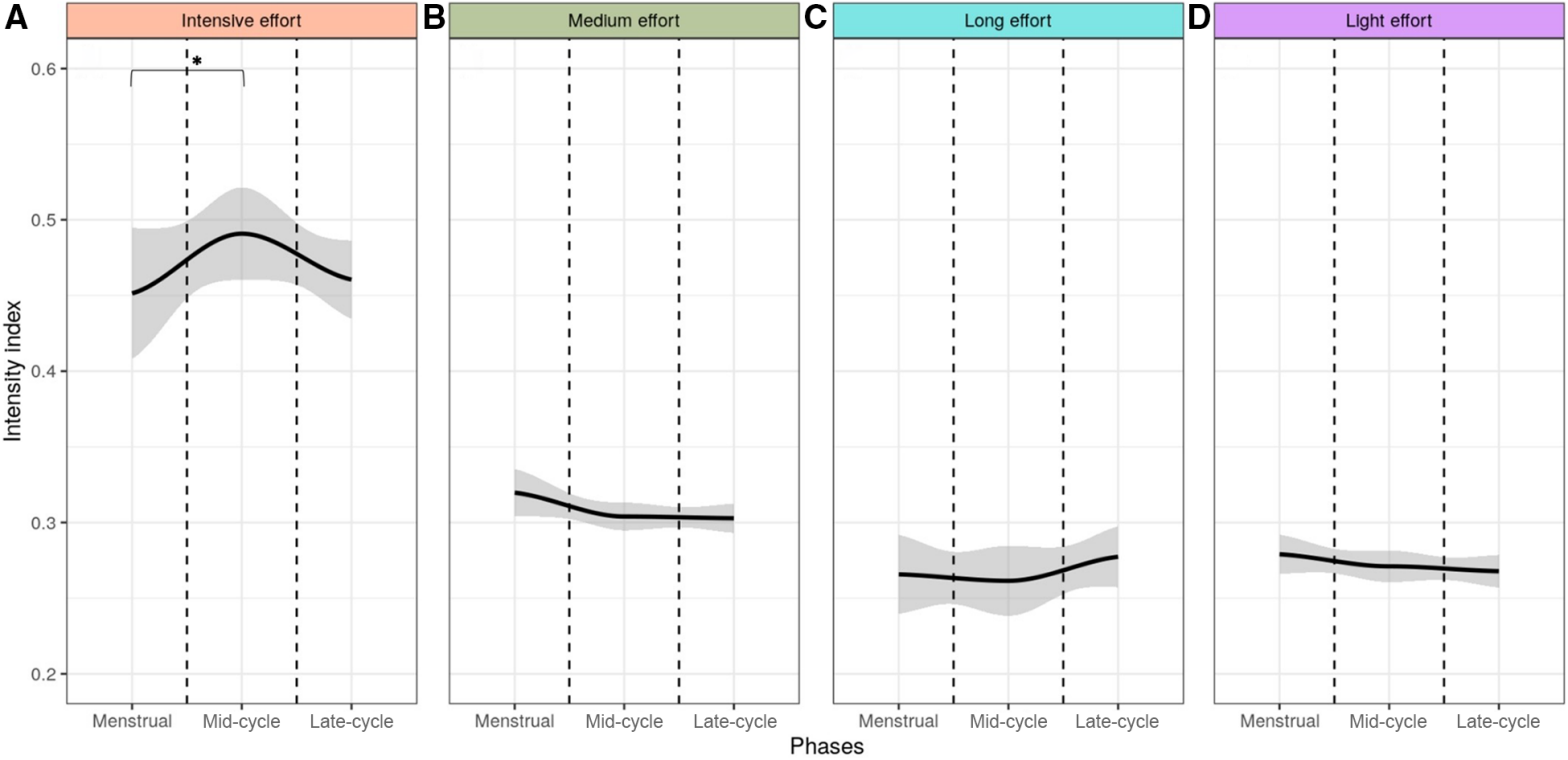
Intensity index across the three menstrual cycle phases among athletes with regular cycle within the intensive (**A**), medium (**B**), long (**C**) and light (**D**) effort clusters. The gray area indicated the 95% confidence interval. *: *p* < 0.05.

**Figure 6:**
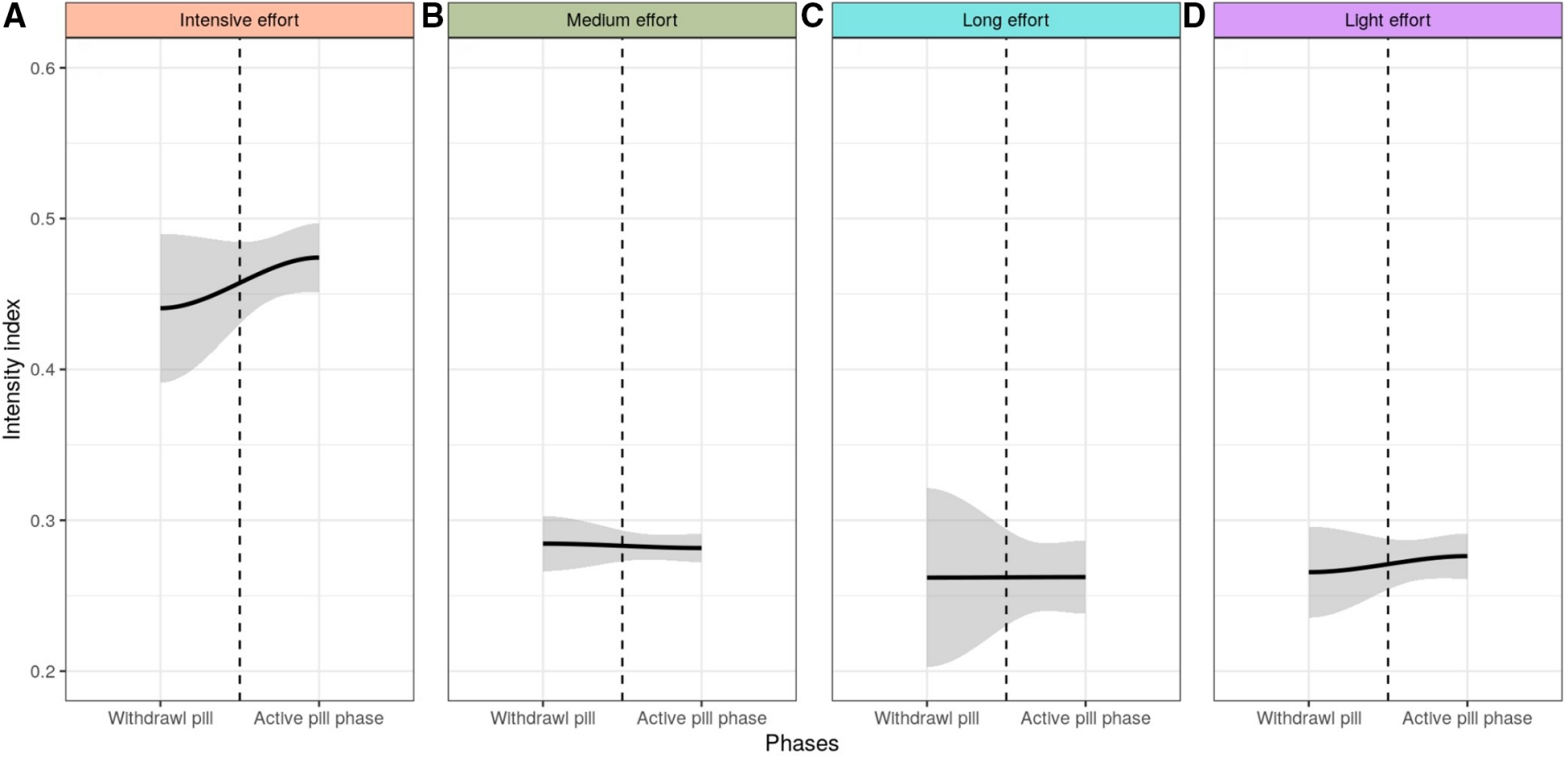
Intensity index across the two oral-contraception phases among among OC-users within the intensive (**A**), medium (**B**), long (**C**) and light (**D**) effort clusters. The gray area indicated the 95% confidence interval.

Within the cluster of intensive effort, we observed a significant difference (*p* = 0.032) in the training intensity index across the estimated MC phases, with a greater intensity index during the MP (0.49 ± 0.03) in comparison to the menstrual (0.45 ± 0.04) phase (Figure 5A), among athletes in the MC group. The LP showed an Intensity index of (0.46 ± 0.025). No significant differences (*p* > 0.05) related to the training intensity were found across the menstrual cycle phases in the other clusters (Figures 5B–D) or among athletes using OC (Figures 6A–D).

## 4 Discussion

This study introduces a novel approach to classify training sessions in cycling according to its intensity, enabling the examination of workload covariates within training sessions with similar demands. It offers valuable insights for monitoring athletes' sessions by providing an intensity index, and a type of training demand assigned by clusters. Relying on this approach this study unveils a peak of training intensity during the mid-cycle phase within the most intensive types of training. Such differences were not found among OC users, or less intensive types of effort.

Relying on multiple variables frequently used to quantify training load in cycling (10, 26), we identified four types of training among 5,190 elite cyclists training sessions, based on their demands in term of both volume and intensity. Similar methods have been used in other sports such as swimming (27). However, they used these methods to identify four groups of subjects according to their training responses, instead of trying to classify the types of training. To the best of our knowledge such training classification has never been done in cycling before. We observed an uneven distribution of training types, with medium efforts being the most common (40%) and intensive efforts being less frequent (10%). Even though this is a new training type classification, differing from previous methodologies, these proportions are concordant with a recent review on training periodization in trained cyclists (28).

In terms of external load variables, the comparison between clusters shows that speed and power are, on average, 50% higher in the intensive effort cluster compared to other training types. The internal load (e.g., RPE and heart rate) reflects this higher demand (29). Similarly, time and distance are, on average, 50% higher in volume efforts compared to other types of training, providing empirical support for the cluster classification, particularly for the most distinct ones, that is, the intensive and long efforts.

When analyzing the intensity demand in each cluster across the estimated MC or OC phases, our study evidences a MC impact when the sessions are classified as the most intensive. Accordingly, in the intensive effort sessions, the intensity index peaks during the middle of the MC, being 8% higher compared to the menstrual phase. It is also 6% higher than the late-cycle days although this difference is not statistically significant. These findings suggest that the athletes' response to training load are influenced by the MC, following an inversed “U-shape”, where the intensity peak is reached in the mid-cycle, especially during the most intensive sessions. It is noteworthy that the coaches' training planning was established in advance and not tailored to the cyclists' phases. Consequently, the various training effort types were distributed randomly across the cyclists' moment in their cycle. Furthermore, most trainings are the same for different cyclists on the same team, while they are on different cycle's days. Hence, the findings of increased intensity index in the training sessions planned to be highly intense show a better training response on mid-cycle.

The different hormonal milieu across the MC could underlie such findings. Female sex hormones fluctuations, including estrogen and progesterone, impact many physiological, psychological and biomechanical systems. The mid-cycle phase, characterized by an increase of estrogens level, and the late-cycle phase characterized by a peak of progesterone combined with a second rise up of estrogen, are associated with specific effects on substrate metabolism, energy store, muscles and perceptual responses (30–33). Given the absence of validated ovulation or hormone level measurements, we can only refer to the mid-cycle as a proxy for the follicular phase and the late phase as a proxy for the luteal phase. Some studies evidenced a difference in glycogen and fat storage or utilization between follicular and late-cycle phase (34). Matsuda et al., observed a greater glycogen utilization in follicular (vs. late-cycle) phase (1). Despite of glycogen utilization, high intensity capacities relies also on neuromuscular parameters, fatigability and recovery. Some studies suggest that neuromuscular function and fatigability change across the MC with a higher voluntary activation and lower fatigability during the late mid-cycle phase (35). Concerning recovery, ventilatory parameters seem to be better in late mid-cycle phase vs. late-cycle phase (36). These metabolic and muscular specificities could support our results, especially the ability to produce higher intensity in the mid-cycle during an intensive training session, corresponding to the cluster of intensive effort.

Recent reviews and meta-analysis reported conflicting and inconclusive results about the impact of menstrual cycle phases on various parameters, especially due to the differences in terms of population, protocol, and statistical analysis (3). The lack of robust research using comprehensive and longitudinal data may explain why previous studies have not consistently demonstrated this impact. Additionally, most studies have not thoroughly explored parameters related to high intensity, which are the specific parameter where our study shows an influence of the estimated MC phases.

Interestingly, a previous longitudinal study relying also on data collected *in situ* during football competitions, when the intensity is at its highest, show similar results (37). The authors evidenced a peak of sprints and high velocity movements in the games during the late mid-cycle phase in comparison to both menstruation and the late-cycle phase.

A recent review suggests that some psychological and perceptual responses are affected in different MC phases. More precisely, a “favorable” subjective response in athletes (i.e., higher motivation, lower RPE) was noticed when the estrogen concentration increases compared to phases with lower concentration (33). Therefore, cyclists could be able to develop more intensity in mid-cycle phase having more motivation and perceiving effort easier. In addition, menstrual symptoms experienced by the cyclists may hinder their ability to generate high intensity efforts and thus partly explain the lower intensity index found in menstrual phase late-cycle phase. It is possible that coaches made some last-minute adjustments to their pre-planned sessions upon noticing a change in a cyclist's readiness, typically when the cyclists display decreased fitness.

For cyclists experiencing menstrual symptoms, it can be potentially easier to train for a longer time at low to moderate intensity during these days (38), since no significant differences were found in long, medium or light efforts clusters. These findings reinforcing that the MC only affect training sessions requiring an effort close to the athlete's maximum capacity. The impact of the MC being evidenced only on most intensive types of elite training could partially elucidate current controversial results in researches, knowing that investigations on this subject have been performed in different types of efforts and level of athletes. In addition, these findings could guide new approaches for studying the MC effect on elite athletes.

No significant differences were found in the intensity index between phases of OC use in any of the four effort types. OC utilization induces a significant down-regulation of endogenous sex hormones (via inhibition of gonadotropic hormones) which may have a potential effect on physiological and psychological processes (39). Our finding is consistent with a recent review which concluded that the exercise performance was consistent across the OC phases (40). Yet, the increased trend in the intensity index in the most intensive effort displayed by the OC-users during the pills' taking phase should be further investigated. In addition, previous studies showed that the OC-users evaluate their training performance lower during the withdrawal phase (17).

### 4.1 Strength and limitations

Despite the robustness of the proposed model, we acknowledge some limitations. Firstly, to perform the trainings classification, it is necessary to have measures of all variables concomitantly to validate the monitoring process. If any of the variables used in the model were missing, the session could not be included in the monitoring as it would not be comparable to the others. Thus, the model requires complete data for all the 14 load variables to generate accurate results. Additionally, as an unsupervised model, it relies on a large volume of data to accurately identify similarities or differences and determine distinct clusters. Although we observe training sessions straddling some clusters, the Silhouette score indicate that sessions well matched to their own cluster. Thus, this streamlined approach provides a more efficient and practical means of categorizing athletes training sessions.

We recognize the potential bias in determining the MC based solely on the calendar method. Indeed, based on the latest recommendations surrounding MC (4, 41), our model and analysis would gain accuracy in using urinary ovulation testing and blood hormonal analysis in order to avoid a mis-categorization between follicular and late-cycle phases and inclusion of anovulatory cycles (19). But it's not practical for elite athletes to use these tests on a regular basis for a longitudinal approach. The potential inclusion of mis-categorized cycles in our research likely diminishes the accuracy of phase divisions, reducing the significance of our results rather than altering their direction. However, the extensive longitudinal monitoring *in situ* of elite athletes represents a notable strength of this study, which enhances the robustness of the proposed model. Further research is needed to address these limitations and refine the model, including the use of larger datasets and incorporating ovulation and hormone measurements for more accurate assessments.

### 4.2 Practical application

To our knowledge, no previous studies in elite cycling have employed such a comprehensive process to analyze the impact of estimated MC or OC phases, making this study unique in its approach. Our model allows to enhance coaches' visibility on athletes' training intensity response across their cycle. This could also be used to analyze other complex physiological parameters affecting training responses since it can be easily integrated in commonly used athlete monitoring systems. In addition, this model can be used at the individual level, either for male or female athletes, which is relevant in elite level, especially in view of the high inter-variability among athletes. If significant individual variations are observed, such as the 8% difference observed here, scheduling high intensity sessions during the “optimal” phase, when the athlete are more naturally prone to perform higher intensity effort, could lead to better training responses. If the pattern of a peak intensity is identified at the individual level, it could be interesting for elite level with regular menstrual cycle to place more intensive training sessions just after the menstruation phase to expect more substantial adaptations to intensive effort, while placing more light-to-moderate sessions during menstruation.

Further research regarding the hormonal phases impact on various performance parameters are necessary to deepen our understanding and enhance training strategies in elite cycling. As evidenced from this study, detecting performance disparities across the MC would probably be easier in the context of exceedingly high constraints. Consequently, future investigations should prioritize this criterion to ascertain physiological effects of MC in elite athletes.

## 5 Conclusion

The clustering model developed provides a training classification according to the intensity load performed by elite cyclists. Since this model allows a multiparametric analysis of massive data collected longitudinally *in situ* it provides a robust framework to investigate the influence of complex variables such as the estimated menstrual cycle phases or the oral-contraception phases. Relying on this approach we showed a peak of training intensity during the mid-cycle, attesting a menstrual cycle effect among the most intensive training sessions. We have not observed a significant impact of the MC in less intensive types of trainings, suggesting an impact of the MC only when the athlete approaches her maximal intensity. No effect of the oral contraception phases was observed in any type of training effort. The fluctuation in cyclists' intensity response evidenced across de estimated MC phases can be a parameter for tailoring and proactively adjusting training plan at individual basis when needed.

## Data Availability

The original contributions presented in the study are included in the article/Supplementary Material, further inquiries can be directed to juliana.antero@insep.fr.
